# Hepatitis B and C virus infections among patients with end stage renal disease in a low-resourced hemodialysis center in Vietnam: a cross-sectional study

**DOI:** 10.1186/s12889-015-1532-9

**Published:** 2015-02-27

**Authors:** Cuong Minh Duong, Dariusz Piotr Olszyna, Mary-Louise McLaws

**Affiliations:** School of Public Health and Community Medicine, UNSW Medicine, UNSW Australia, Level 3 Samuels Building, Sydney, NSW 2052 Australia; Division of Infectious Diseases, University Medicine Cluster, National University Health System, Singapore, Singapore

**Keywords:** Hepatitis B virus, Hepatitis C virus, Hemodialysis, End stage renal disease, Vietnam, Universal precautions

## Abstract

**Background:**

Hemodialysis services in Vietnam are being decentralised outside of tertiary hospitals. To identify the challenges to infection control standards for the prevention of bloodborne infections including hepatitis B virus (HBV) and hepatitis C virus (HCV) we tested the magnitude of HBV and HCV infections in the largest unit in Ho Chi Minh City servicing patients with end stage renal disease.

**Methods:**

All 113 patients provided consent HBV surface antigen (HBsAg) and HCV core antigen (HCV-coreAg) testing. Positive patients were tested for viral genotypes. All participants completed a questionnaire on demographic characteristics, risk factors and previous attendance to other hemodialysis units.

**Results:**

Seroprevalence of 113 patients enrolled was 7% (8/113, 95% CI 2.3%-11.8%) HBsAg, 6% (7/113, 95% CI 1.7%-10.6%) HCV-coreAg and 1% (1/113, 95% CI 0.8%-2.6%) co-infection. Having a HBV positive sexual partner significantly increased the risk of acquiring HBV (P = 0.016, Odds Ratio (OR) =29, 95% CI 2–365). Risk factors for HCV included blood transfusion (P = 0.049), multiple visits to different hemodialysis units (P = 0.048, OR = 5.7, 95% CI 1.2–27.5), frequency of hemodialysis (P = 0.029) and AST plasma levels >40 IU/L (P = 0.020, OR = 19.8, 95% CI 2.3–171). On multivariate analysis only blood transfusion remained significant risk factor for HCV (P = 0.027, adjusted OR = 1.2).

**Conclusions:**

HCV screening for HCV of blood products must improve to meet the infection prevention challenges of decentralizing hemodialysis services. The level of HCV and HBV in our hemodialysis unit is a warning that universal precautions will be the next challenge for decentralised hemodialysis services in Vietnam.

## Background

Hemodialysis is routinely used as renal replacement therapy for end stage renal disease (ESRD) patients [1]. In 2012, 2.1 million patients worldwide were estimated to require hemodialysis and this number is expected to increase by 7% annually [2]. Chronic hemodialysis patients are at increased risk for both HCV and HBV infections associated with contaminated blood and blood product transfusion and exposure to contaminated hemodialysis equipment during treatment [3-6]. Like the community, chronic hemodialysis patients may also acquire HBV and HCV through other at-risk activities, such as unprotected sexual contact and injecting drug use [7,8]. Several infection prevention strategies that are effective in reducing the acquisition of bloodborne virus by patients in high resourced healthcare settings includes erythropoiesis-stimulating agents which has reduced the need of blood transfusion, HBV vaccination and the adherence by hemodialysis services to specific hemodialysis infection control guidelines [9].

In 1983 Vietnam commenced its hemodialysis services at tertiary hospitals for ESRD patients [10]. In Vietnam 6 million, that is 6.73% of the general population, have been estimated to be diagnosed with chronic kidney disease [11]. Of these 6 million patients 80,000 (1.3%) patients have already reached ESRD. Currently, the healthcare system in Vietnam can only provide treatment to 8,000 (10%) of patients with ESRD. Further, 8,000 patients will be newly diagnosed annually of whom 104 (1.3%) will also go onto require hemodialysis services of who 10 will receive hemodialyis [11]. To address the growing demand for hemodialysis, health services will be decentralised out of tertiary hospitals and become provincial services. Routine universal precautions and infection control practices for the prevention of bloodborne infections need to be established to protect the growing number of ESRD patients from further morbidity. Our study hemodialysis unit was the first decentralised district unit established in Ho Chi Minh City in Southern Vietnam in 2009. Since 2009 a further seven units now offer services to an average of 60–70 patients. We report our preliminary findings of the point seroprevalence, risk factors and genotype of HBV and HCV in newly admitted ESRD patients.

## Methods

### Design of the study

In the period between October 2012 and January 2013 all patients aged 18 years and older, newly admitted at the Hemodialysis Unit, District-6 Hospital, Ho Chi Minh City, Vietnam were enrolled to participate at the study. The study protocol was approved by the Human Research Ethics Committee at UNSW Australia (approval number HC12363), the Ho Chi Minh City Health Service and District-6 Hospital authorities.

Participants completed a self-administered questionnaire that comprised of demographics (age and sex), risk factors that included unprotected sexual contact, injecting drug use, barber use of razor blade shaving, piercings, tattooing, blood and blood product transfusions, and attendance to different hemodialysis units. Participants provided location, name of the hemodialysis unit and the year of admission so that the data for the number of different hemodialysis unit visits could be assessed as a risk factor. Assistance with the questionnaire was provided when needed by one investigator. Participants consented to HBV and HCV serology testing, viral genotyping. Aminotransaminases (AST and ALT), y-glutamyl transpeptidase (GGT), medical and dialysis information were obtained from patients’ medical records. Dialysis information included: history of blood transfusion, surgery, abnormal liver function, duration (in months) of having hemodialysis services, frequency (in days) of hemodialysis treatments, number of different hemodialysis units admitted for hemodialysis services and dialyzer reuse.

To validate the information of self-reported transfusion history all medical records were reviewed for accuracy. Patients who reported a blood transfusion prior to admission to the unit were asked to provide the year, place of transfusion and the number of transfusion sessions.

HBV vaccination of new patients is currently not routinely practiced. Patients are encouraged to have HBV vaccination at their own cost. None of our patients is vaccinated. Financial constraint means it is common practice in Vietnam to reuse dialyzers. Used dialyzers are labeled with the patient’s name. Used dialyzers for each patient, regardless of infection status, are cleaned and stored separately for reuse by the original identified patient. Given the unavailability of dedicated room for HBV or HCV infected persons, the study unit has designated machine for HCV or HBV infected patients. The unit plans weekly dialysis schedules according to patient availability and by infection status. Infected patients are always planned to receive their treatments at less crowded dialysis sessions. Patients are required to follow their dialysis schedules so they receive treatment at a given dedicated dialysis machine at a fixed time.

### Laboratory tests

Serum samples obtained by centrifugation of blood samples at 3,000 g for 10 minutes were stored at −20°C prior to testing. The ARCHITECT HCV Ag assay (ABBOTT Laboratories) which is a Chemiluminescent Microparticle Immunoassay (CMIA) was used to detect HCV-coreAg [12,13]. The ARCHITECT HBsAg assay (ABBOTT Laboratories), another CMIA, was used to detect the presence of HBsAg [14]. Positive samples using these assays were tested for HBV and HCV genotype by TRUGENE HBV Genotyping kit [15] and TRUGENE HCV 5′NC Genotyping kit (SIEMENS) [16,17] respectively. Immunoassays did not require sample processing. For HBV genotyping, DNA from serum samples extracted by MagNAPure LC instrument (Roche Diagnostics) was amplified before performing Crosslinking and immunoprecipitation (CLIP) sequencing to obtain the query sequence in accordance with the manufacturer’s protocol. Genotype was identified by comparing the query sequence to the HBV genotype consensus sequences of the OpenGene system software. For HCV genotyping, a 244-base pair sequence in the 5′non-coding region of HCV RNA extracted by MagNAPure LC instrument (Roche Diagnostics) was amplified before performing bidirectional CLIP sequencing to obtain the sequencing products. These segments were then detected using the OpenGene DNA Sequencing System. The forward and reverse sequences were combined to form a query sequence. Genotype was identified by comparing the query sequence to the previously characterized isolates in the Trugene HCV 5′NC Molecule of the OpenGene system software.

### Statistical analysis

Data were managed and analysed using Statistical Package for the Social Sciences (SPSS) version 20 (IBM). Fisher’s Exact test was used to analyse the significant relationships between categorical data and Mann–Whitney *U* test was used for the comparison of continuous data. Correlations were used to test for the strength of association between continuous variables. A multinomial logistic regression (MLR) model was used to test risk factors for HCV seroconversion. Independent variables were entered into the model and included quantity of blood transfusion, number of different hemodialysis units admitted for treatment, the frequency of hemodialysis treatments, duration of hemodialysis, and AST >40 IU/L. A HBV seroconversion model to identify significant risk factors could not be built. Alpha was set at 5% level.

## Results

### Predisposing factors

**Figure 1:**
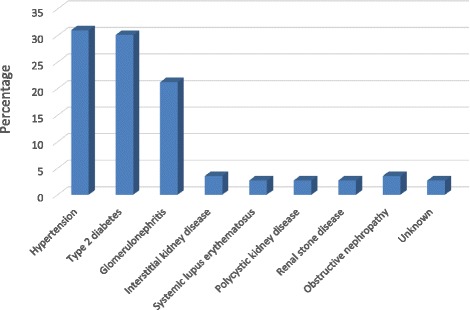
**Etiologies of end stage renal disease on admission.**

113 participants attended the clinic during the 4 months of enrolment. The mean age of participants was 53 years (SD ± 16 years, range 18–86 years) (Table 1). Just over half (52%) of the participants were female and most (98%) reused dialyzers. The mean duration of hemodialysis was 36 months (SD ± 43 months, range 1.8–245.5 months). The mean number of hemodialysis treatments was 391 (SD ± 489, range 7–2946 events) and 99% (112/113) were documented to have received treatment at other hemodialysis units. Over half (65%, 73/113) of all patients had received a blood transfusion. Causes of ESRD included hypertension (31%, 35/113), type-2 diabetes mellitus (30.1%, 34/113), glomerulonephritis (21.2%, 24/113), obstructive nephropathy plus interstitial kidney disease (3.5%, 4/113) and unknown cause (2.7%, 3/113) (Figure 1).

**Table 1:** **Patient demographics and clinical characteristics**

Female % (n/N)	52 (59/113)
Age (years)	
Median [LQ; UQ]	54 [40; 64]
(Mean ± SD)	(53 ± 16)
Range	18–86
Duration of hemodialysis (months)	
Median [LQ; UQ]	18 [10; 38]
(Mean ± SD)	(36 ± 43)
Range	1.8–245.5
Frequency of hemodialysis treatments (days)	
Median [LQ; UQ]	195 [87; 441]
(Mean ± SD)	(391 ± 489)
Range	7–2946
Patients reusing dialyzer % (n/N)	98 (111/113)
Patients previously having blood transfusion % (n/N)	65 (73/113)
Patients having different hemodialysis unit visits % (n/N)	99 (112/113)

### Seroprevalence and genotyping

Most patients (86%, 95% CI 79.4%-92.2%, 97/113) were negative for both HBsAg and HCV-coreAg, 7% (95% CI 2.3%-11.8%, 8/113) were HBsAg positive, 6% (95% CI 1.7%-10.6%, 7/113) were HCV-coreAg positive and 1% (95% CI 0.8%-2.6%,1/113) was HBsAg and HCV-coreAg positive. The viral genotype distribution among patients who were HCV-coreAg positive was 1b (3 patients), 1a (2 patients) and 6a (1 patient) and 2 patients who were HCV-coreAg positive with untypable virus. There were 5 patients with HBV genotype B and 4 patients who were HBsAg positive with untypable virus.

### Risk factors for HCV infection

Five risk factors (unprotected sexual contact, injecting drug use, barber use of razor blade shaving, piercings and tattooing) were not significantly associated with HBV or HCV.

None of the HCV positive patients reported to have HCV-positive sexual partner (Table 2). Patients who were HCV positive were significantly more likely have received at least one blood transfusion (P = 0.049) and significantly more likely (P = 0.048, OR 5.7, 95% CI 1.2-27.5) to have visited more than two different hemodialysis units compared with HCV negative patients. The amount of blood unit transfused was found to be significantly correlated with duration of hemodialysis (r = 0.33, p < 0.01). HCV positive patients had significantly more hemodialysis treatments than HCV negative patients (949 vs 348, P = 0.029) (Table 3). The duration of hemodialysis was not a significant risk factor (P = 0.068).

**Table 2:** **Unadjusted risk factors tested for hepatitis C and hepatitis B infections**

**Risk factors**	**HCV % (N)**			**HBV % (N)**		
	**Positive (8)**	**Negative (105)**	**P** ^**a**^	**Positive (9)**	**Negative (104)**	**P** ^**a**^
HBV-positive sexual partners	0	2.8 (3)	undetermined OR 1.0	22.2 (2)	1 (1)	29 (2–365) 0.016
HCV-positive sexual partners	0	1.0 (1)	undetermined OR 1.0	11.1 (1)	0	undetermined OR 0.08
At least one blood transfusion	100 (8)	61.9 (65)	undetermined OR 0.049	77.8 (7)	63.5 (66)	2.0 (0.4-10.2) 0.5
Having >2 different hemodialysis unit visits	37.5 (3)	9.5 (10)	5.7 (1.2–27.5) 0.048	11.1 (1)	11.5 (12)	1.0 (0.1-8.3) 1.0

^a^Fisher’s Exact Test.

**Table 3:** **Association between hemodialysis treatment and HCV and HBV infections**

**Risk factor**	**HCV (mean ± SD)**			**HBV (mean ± SD)**		
	**Positive (N = 8)**	**Negative (N = 105)**	**P** ^**b**^	**Positive (N = 9)**	**Negative (N = 104)**	**P** ^**b**^
Frequency of hemodialysis treatments (days)	949.4 ± 998.5	348 ± 405.5	0.029	366.1 ± 251.9	392.7 ± 504.9	0.284
Duration of hemodialysis (months)	80.1 ± 82.6	32.6 ± 37.2	0.068	32 ± 20	36.3 ± 44.6	0.351

^b^Mann-Whitney *U* test.

### Risk factors for HBV infection

Patients with HBV positive sexual partners were significantly more likely (P = 0.016, OR 29.0, 95% CI 2.0-365.0) to be HBV positive compared HBV negative patients (Table 2). Visiting more than two different hemodialysis units (P = 1.0), duration of hemodialysis treatment (P = 0.351) (Table 3) and the frequency of hemodialysis treatments (P = 0.284) were not a risk factor for HBV infection.

### Association between AST, ALT and GGT and HBV and HCV infections

After removing potential risk factors (cirrhosis, alcohol and herbal consumption) that can increase liver enzyme, ALT, AST and GGT >40 IU/L were observed in 3% (3/113), 4% (4/113) and 23% (25/113) of study participants respectively. AST >40 was associated with HCV positivity (P = 0.02, OR 19.8, 95% CI 2.3-171) while ALT >40 (P = 0.18) and GGT >40 (P = 0.35) were not. AST, ALT and GGT >40 were not associated with HBV positivity (P > 0.05).

### Model for the prediction of seroconversion

No other predictor for HCV seroconversion was identified other than the number of blood transfusion (AOR 1.16, 95% CI 1.01-1.33, P = 0.027) (Table 4).

**Table 4:** **Multinominal logistic regression analysis for predictors of HCV infections**

**Risk factors**	**P**	**Adjusted OR (95% CI)**
Number of transfused blood (unit)	0.027	1.16 (1.01–1.33)
Number of different hemodialysis unit visited	0.35	1.70 (0.57–5.05)
Frequency of hemodialysis treatments (days)	0.33	1.01 (0.99–1.03)
Duration of hemodialysis (months)	0.36	0.93 (0.74–1.17)
AST >40 (IU/L)	0.13	7.20 (0.65–79.94)

## Discussion

The seroprevalence in hemodialysis patients in Western countries ranges from 5%-10% for HCV and 0%-6.6% for HBV [9,18]. The seroprevalence rates in our patients were similar at 6% for HCV and 7% for HBV. Anti-HCV and HBsAg assays are the usual method for screening blood donors in Southern Vietnam where our study was conducted. The HCV screening rate in Southern Vietnam blood donors is reported at 0.8%, 7.5 times smaller than the rate in our patients and the HBV screening rate in donors, 3.7%, is also less than the rate we found in our patients [19]. The implementation of infection control precautions were issued in 1997 in Vietnam [20]. However, prior to 2001, the prevalence rates for HCV among chronic hemodialysis patients in tertiary hospitals in Ho Chi Minh City and Hanoi, Vietnam, were hyper-endemic at 44% to 57% respectively [21,22]. Gradually awareness of infection control measures for blood transfusion safety increased and screening all blood products for bloodborne viruses commenced in 2001 [23]. In 2009 the prevalence of HCV in hemodialysis patients from 5 major cities across Vietnam had reduced but remained hyper-endemic at 26.6% [24]. A six-year cohort study, from 2006 to 2011, in one hemodialysis unit in Ho Chi Minh City the HCV prevalence of 19.6% (95% CI 12.4%–15.2%) was three times higher than the prevalence in our hemodialysis patient population [25]. Long-term hemodialysis patients may induce false-negative results as a result of an unstable immune response in up to 17.9% of patients tested [26]. We believe the false negative results in our study will have been negligible as the specificity of HCV-coreAg assay was 99.8% and the sensitivity of the assay is equivalent to HCV-RNA assay [12]. The prevalence for HBV at 4.9% (95% CI 2.2%–7.2%) [25] and co-infection of HCV and HBV at 2.1% (95% CI 1.0%–3.3%) [25] from the cohort study were similar to the rates in our population. Currently, universal precautions are applied in Vietnam. Yet, our findings suggest universal precaution guidelines have yet to become routine practice. In addition, seven of our eight HCV positive patients were transfused after 2001. The hyper-endemic HCV and HBV in our hemodialysis patients suggests that poorly screened blood products are likely to be the source of infection. Besides, age at the time of acquiring HBV infection is strongly associated with population endemicity [27]. Vietnam is a high HBV endemic area [28,29] with perinatal and horizontal routes still responsible for the majority of transmission [27].The rate of HBV in our patient population was similar to other hemodialysis patients in Vietnam several years earlier [25] suggesting the risk is stable possibly due to the high level of community infection acquired early in life. Hemodialysis patients in Vietnam would benefit from HBV vaccination prior commencing hemodialysis treatment.

Blood transfusion is an important risk for HCV infection among maintenance hemodialysis patients [18]. Our HCV positive patients were more likely to have acquired infection from multiple clinics and receiving more than two blood transfusions. The quality and type of diagnostic test used will impact on the false negative rates making comparisons of prevalence infection prevention strategies between countries and in-country units inaccurate. The duration of the window period, the time between the onset of HCV infection and first detection of antibodies/antigens in the bloodstream, varies with the most commonly used generation of serology test from 66 days to only 4 days with the nucleic acid test (NAT) [30]. The NAT test has greater sensitivity for detecting HCV. Several high resourced countries since 1997 have used NAT as the routine screening test for blood donors [31]. By 2015 major cities in Vietnam will use NAT as the screening test for blood donations [32]. We found a modest but significant linear relationship between blood transfusions and prolonged hemodialysis treatment (r = 0.33, p <0.01). The decision to transfuse ESRD patients with anemia is dependent on several indicators, the patient’s age, presence of cardiovascular diseases and severity of anemia symptoms [33]. A third of our patients were ≥60 of age, 43% had cardiovascular diseases and a quarter had chronic blood loss due to gastrointestinal diseases. These indications support repeated blood transfusion and the use of erythropoiesis-stimulating agents. However, one unit of blood transfusion increased the odds for HCV in our patients by 1.16 times. Yet, by strictly complying with a treatment plan, patients can reduce the risk of anemia that requires blood transfusions and possible HCV infection. Time on hemodialysis was not a risk factor for HCV infection in our study though it was reported elsewhere [18,34]. This can be explained by the fact that it may not reflect the true duration of exposure to hemodialysis. Financial constraints often prevent patients in Vietnam from following their treatment plan. The risk associated with the frequency of hemodialysis treatments and HCV could therefore be a proxy for duration.

Challenges for patients having multiple hemodialysis treatments include repeated vascular access [6] and potential for exposure to contaminated venous access devices due to breaches in infection control precautions [35]. Increased risk for HCV was associated with visits to multiple hemodialysis units in our study and elsewhere [36] which suggests differences in infection control precautions occur between units. In Vietnam, arteriovenous fistula (FAV) operation is not routinely performed outside a tertiary urban unit. Patients from smaller units found outside the larger cities, are currently referred to tertiary units for FAV operation. During their admission for FAV the patient will receive hemodialysis. Nearly all our patients had visited at least two other hemodialysis units. Smaller units may one day provide FAV surgery to reduce the cost and inconvenience to patients who are forced to be referred to units in the city. But the practice of universal precautions will need to be routinely applied to prevent surgery-associated infections.

HCV genotypes and subtypes are geographically diverse [37,38]. In Vietnam, type 1b and 6a are the predominant genotypes [39,40]. Among the typable infections in our patients, HCV genotype 1b was predominant followed by 1a and 6a. Sixty-four chronic hemodialysis HCV infected patients from Northern Vietnam in 2008 were found to have a similar genotype distribution with predominance of genotype 1 [41]. But the real-time RT-PCR technique used in this study prevented the HCV subtype from being identified. Among the typable HBV infections, the most common genotype was B concurring with reports from other patient sources in Vietnam [42,43].

Increased transaminase levels are associated with HCV infection [44]. However, elevation of transaminases (>40 IU/L) is not always present in chronic hemodialysis patients with viral hepatitis [45]. We found that AST >40 was significantly associated with HCV infection. This suggests that an unexplained or slight increase in AST could be used to signal early HCV seroconversion. We did not find an association between increased transaminases and HBV infection.

## Conclusions

The challenge for Vietnam as it rolls out hemodialysis units from teaching hospitals into smaller centers will be the faultless routine application of universal precautions, early HBV vaccination and stringent screening of blood products countrywide.

## Abbreviations

ALT: Alanine aminotransferase
AST: Aspartate aminotransferase
FAV: Arteriovenous fistula
CMIA: Chemiluminescent microparticle immunoassay
ESRD: End stage renal disease
GGT: y-glutamyl transpeptidase
HBV: Hepatitis B virus
HCV: Hepatitis C virus
HBsAg: Hepatitis B virus surface antigen
HCV-coreAg: Hepatitis C virus core antigen
NAT: Nucleic acid test
OR: Odds ratio
UNSW: University of New South Wales
